# Secondary Postoperative Hemorrhage in the Pediatric Tonsillectomy Patient- is there a correlation between hemorrhage and tonsilloliths?

**DOI:** 10.51894/001c.57320

**Published:** 2023-12-05

**Authors:** Andrew Ross, Ani Mnatsakanian, Jacob Markovicz, Sruti Desai, Brian Anderson, Holly Shifman, Steven Engebretsen, Carissa Wentland, Prasad Thottam, Michael Haupert

**Affiliations:** 1 College of Osteopathic Medicine Michigan State University; 2 Department of Otolaryngology – Head and Neck Surgery Detroit Medical Center; 3 Department of Otolaryngology – Head and Neck Surgery Ascension Macomb-Oakland Hospital; 4 William Beaumont School of Medicine Oakland University; 5 Children’s Hospital of Michigan; 6 Michigan Pediatric Ear Nose and Throat Associates

**Keywords:** Tonsillolith, Post-tonsillectomy hemorrhage, pediatrics, tonsillectomy

## Abstract

**INTRODUCTION:**

Tonsillectomy with or without adenoidectomy is one of the most common ambulatory procedures performed in children under 15. One rare yet serious complication of tonsillectomy is postoperative hemorrhage. Chronic tonsillitis, which is an indication for tonsillectomy, has been shown to have an increased risk for postoperative hemorrhage. Tonsilloliths or tonsil stones have been associated with cryptic tonsillitis. This 2020-2021 study examined whether tonsilloliths were a risk factor for post-tonsillectomy hemorrhage in a convenience sample of 187 pediatric patients.

**METHODS:**

This was a cross-institutional 12-month retrospective cohort study investigating pediatric patients who had undergone tonsillectomy. Exclusion criteria included patients who had received prior airway surgeries (e.g., supraglottoplasty), patients with significant comorbidities such as chromosomal abnormalities or congenital disorders, and patients with pre-existing bleeding disorders. Demographic, clinical, and operative data was extracted from each chart. Postoperative adverse events and bleeding were also recorded. These factors were then compared between the tonsillolith and no tonsillolith patient groups.

**RESULTS:**

A total of 187 pediatric patients met the inclusion criteria. Seventy-three (39%) of the patients had tonsilloliths and 114 (61%) did not have tonsilloliths at the time of surgery. The tonsillolith subgroup had a higher median age (10 vs 3, P < 0.001) when compared to the no tonsillolith subgroup. The most common indication for tonsillectomy was obstructive sleep apnea/sleep disordered breathing (N= 148, 79.1%). There was no statistical difference found between presence of tonsillolith and indication for surgery (P = 0.06). Only five (2.7%) of sample patients experienced postoperative bleeding and there was no association found between postoperative bleeding and presence of tonsilloliths (P = 0.38).

**CONCLUSION:**

In the current study there was no association found between the presence of tonsilloliths (indicating low grade chronic inflammation) and hemorrhage after tonsillectomy. Continued larger sample evaluations of possible risk factors for post-tonsillectomy hemorrhage patterns are encouraged.

## INTRODUCTION

Tonsillectomy with or without adenoidectomy is one of the most common ambulatory surgical procedures performed in the United States in children younger than 15 years of age.^1^ The two most common indications for tonsillectomy include recurrent tonsillitis and obstructive sleep-disordered breathing. Other common indications include recurrent tonsillitis in the setting of multiple antibiotic allergies, PFAPA (i.e., periodic fever, aphthous stomatitis, pharyngitis, adenitis), recurrent peritonsillar abscess, halitosis, and febrile seizures.^2–4^

Although tonsillectomy is considered a straightforward procedure, it has been associated with some significant postoperative complications (e.g., oropharyngeal pain, dehydration, velopharyngeal insufficiency, and hemorrhage from the operation site).^5^ Given the robust vascular supply of the tonsils, postoperative hemorrhage after tonsillectomy can be life threatening. The rate of post-tonsillectomy hemorrhage ranges from 2.1% - 13.4% across all age groups^6–15^ with the rate of secondary surgery to control hemorrhage ranging from 1.2% - 8%.^7,9,14^

Prior studies have suggested that patient age, male gender, low-socioeconomic status and recurrent tonsillitis are risk factors for post-tonsillectomy hemorrhage.^6,9,16^ There has been limited literature to date suggesting that cryptic tonsils and bacterial colonization of the tonsil are significantly associated with increased post-tonsillectomy hemorrhage, however these studies did not specify whether the tonsils contained tonsilloliths perioperatively.^17,18^

Tonsilloliths are calcifications that form within tonsillar crypts and may be found in both children and adults. They often contain aerobic and anaerobic bacteria and act as microbacterial biofilms.^19^ Studies have demonstrated that cultures of these tonsilloliths can grow *Prevotella* spp. and *Fusobacterium* spp, which are similar microbes isolated in acute tonsillitis.^20,21^ Anaerobes isolated from tonsilloliths can produce volatile sulfur compounds which are often the source of halitosis.^22^ Most importantly, tonsilloliths are associated with chronic cryptic tonsillitis, the most common indication for adult tonsillectomy.^23^

The authors’ hypothesis, in conjunction with the published literature and clinical correlations, endorsed that patients with tonsilloliths would have tonsillar tissue in a chronic inflammatory state. Despite its association with chronic tonsillitis, there has been limited discussions on the role of tonsilloliths specifically as a risk factor for post-tonsillectomy hemorrhage. Thus far, no study has apparently examined post-tonsillectomy hemorrhage rates after tonsillectomy in patients with tonsilloliths versus those without them.

### Study Purpose

The aim of this study is to understand whether the association between tonsilloliths, hence a chronic inflammatory state, poses a significantly greater risk for post-tonsillectomy hemorrhage in a convenience sample of postoperative pediatric patients.

## METHODS

After approval from the authors’ three institutional review boards, a retrospective cohort study was conducted of pediatric (i.e., <18 years of age) patients who had received a tonsillectomy between December 1, 2020 - December 31, 2021. This time period was examined as prior to December 1,2020 the operative templates did not include documentation of tonsilloliths. Exclusion criteria included patients who had other airway surgery in addition to the tonsillectomy (e.g., supraglottoplasty), patients with significant comorbidities such as chromosomal abnormalities or congenital disorders, and patients with pre-existing bleeding disorders. Also, if an operative report did not specify the presence or absence of tonsilloliths the patient was excluded from this study.

Surgery was conducted under general anesthesia with orotracheal intubation. Surgical technique included first dissecting the superior pole and then excising the tonsil in its entirety along the peritonsillar space. Any intraoperative bleeding was controlled either with suction cautery (when using monopolar cautery) or with the coagulation setting when using the coblator wand. For pain control, all patients were prescribed weight-based doses of tylenol and ibuprofen. Patients were also prescribed oxycodone when age-appropriate (age >3 years old). All patients were instructed to maintain a soft diet and light activity as tolerated for the first 14 days after surgery. Patients were advised to return to the emergency room immediately if any bleeding occured.

Demographic, clinical, and operative data (age, gender, body mass index (BMI), apnea-hypopnea index (AHI), surgical indication, intraoperative time, adverse events, and presence of tonsilloliths) were collected and analyzed for a sample of 187 patients using in-office patient charts and the electronic health record (EHR). For this study, post-tonsillectomy hemorrhage (PTH) was defined as any patient who presented for PTH even if they were negative upon clinical examination or did not require observation or surgical intervention. Types of hemorrhage were defined as primary hemorrhage (occurring within 24 hours of surgery) or secondary hemorrhage (occurring at least 24 hours after surgery).

### Study Analyses

Continuous data were compared between patients with and without tonsilloliths using Wilcoxon rank sum tests and are reported as medians and interquartile ranges. Categorical data were compared between groups using Fisher’s exact 2-tail tests or Chi-squared tests, as appropriate, and frequencies and percentages are reported. For all measures, statistical significance was accepted if the p-value was < 0.05. All statistical analyses were performed in R (Version 4.1.0, The R Project for Statistical Computing).

## RESULTS

A total of 187 pediatric patients undergoing tonsillectomy at Beaumont Children’s Hospital Royal Oak and Children’s Hospital of Michigan between January 1, 2020, and December 31, 2021 were included in analyses. Seventy-three (39%) of the patients had tonsilloliths and 114 (61%) did not have tonsilloliths at the time of surgery. Baseline demographic and clinical characteristics are listed in Table 1. Eighty-five (45.5%) patients were female, and the median age at the time of surgery was 5 years (interquartile range [IQR]: 3, 9). The median age and BMI were significantly higher in the tonsillolith group (P<0.001, P=0.0001, respectively). The most common indication for tonsillectomy was obstructive sleep apnea/sleep disordered breathing (N= 148, 79.1%). There was no statistical difference between presence of tonsillolith and indication for surgery(P=0.06)

**Table 1.** Characteristics of pediatric patients who underwent tonsillectomy with and without tonsilloliths.

**Characteristic**	**Overall**	**Tonsilloliths**	**No Tonsilloliths**	**P-value**
**N**	187	73 (39.0)	114 (61.0)	
**Age at Surgery (yrs.)**	5.0 (3.0, 9.0)	10.0 (5.0, 14.0)	3.0 (2.0, 6.0)	**<0.001**
**Sex**				0.19
Male	102 (54.5)	35 (47.9)	67 (58.8)	
Female	85 (45.5)	38 (52.1)	47 (41.2)	
**Body Mass Index**	18.2 (15.8, 24.2)	19.8 (16.9, 25.9)	16.9 (15.2, 21.8)	**0.001**
**Preoperative AHI**	8.5 (4.9, 18.0)	8.0 (5.3, 12.3)	9.1 (4.0, 19.9)	0.50
**Indication for Surgery**				0.06
OSA/SDB	148 (79.1)	51 (69.9)	97 (85.1)	
Chronic Tonsillitis	20 (10.7)	12 (16.4)	8 (7.0)	
Both (OSA/SDB and chronic tonsillitis)	4 (2.1)	3 (4.1)	1 (0.09)	
Other	15 (8.0)	7 (9.6)	8 (7.0)	
**Intraoperative Duration (min.)**	26.0 (20.0, 35.0)	25.0 (18.0, 36.0)	27.0 (21.0, 34.0)	0.46
**Intraoperative Adverse Event**	0 (0)	0 (0)	0 (0)	N/A
**Postoperative Adverse Event**	25 (13.4)	8 (11.0)	17 (14.9)	0.58
**Postoperative Bleeding**	5 (2.7)	3 (4.1)	2 (1.8)	0.38

**Legend:** Values are represented as median (IQR) or frequency (%). Continuous variables were compared using Wilcoxon rank sum tests; categorical variables were compared using χ^2^ tests or Fisher’s exact tests. Empty cells in the p-value column represent comparison across all categories of a variable.

**Abbreviations:** IQR, interquartile range; OR odds ratio; 95% CI, 95% confidence interval; AHI, apnea hypopnea index; OSA, obstructive sleep apnea; SDB, sleep disordered breathing.

There were no patients who experienced intraoperative adverse events, 25 patients (13.4%) who experienced postoperative adverse events (e.g., decreased oral intake, uncontrolled postoperative pain, hypoxic events), and 5 (2.7%) who experienced postoperative bleeding. There were no associations found between the presence of tonsilloliths and adverse events or postoperative bleeding (Table 2). There were also no significant associations identified between any demographic or clinical variables and postoperative adverse events or bleeding. Finally, there was no difference in operative times for the two cohorts studied.

**Table 2.** Association of demographic and clinical characteristics of pediatric tonsillectomy patients with postoperative bleeding and postoperative adverse events.

**A. Postoperative Bleeding**			
**Characteristic**	**Postoperative Bleeding**	**No Postoperative Bleeding**	**P-⁠value**
**N**	5 (2.7)	182 (97.3)	
**Age at Surgery (yrs.)**	6.0 (4.0, 11.0)	5.0 (3.0, 9.0)	0.60
**Sex**			
Male	2 (40.0)	100 (54.9)	0.66
Female	3 (60.0)	82 (45.1)	
**Tonsilloliths Present**	3 (60.0)	70 (38.4)	0.38
**Body Mass Index**	15.3 (14.8, 18.9)	18.3 (15.8, 24.5)	0.23
**Preoperative AHI**	11.0 (9.5, 12.5)	8.5 (4.5, 18.8)	0.72
**Intraoperative Duration (min.)**	25.0 (23.0, 27.0)	26.0 (20.0, 35.0)	0.66
**B. Postoperative Adverse Event(s)**			
**Characteristic**	**Postoperative Adverse Event**	**No Postoperative Adverse Event**	**P-value**
**N**	25 (13.4)	162 (86.6)	
**Age at Surgery (yrs.)**	4.0 (2.0, 6.0)	5.0 (3.0, 9.8)	0.10
**Sex**			
Male	17 (68.0)	85 (52.4)	0.22
Female	8 (32.0)	77 (47.5)	
**Tonsilloliths Present**	8 (32.0)	65 (40.1)	0.58
**Body Mass Index**	17.8 (15.2, 22.3)	18.5 (15.9, 24.4)	0.70
**Preoperative AHI**	8.3 (6.3, 13.4)	8.9 (4.5, 18.8)	0.91
**Intraoperative Duration (min.)**	25.0 (21.0, 33.0)	26.0 (20.0, 35.0)	0.86

**Legend:** Values are represented as median (IQR) or frequency (%). Continuous variables were compared using Wilcoxon rank sum tests; Categorical variables were compared using Fisher’s exact tests (postoperative bleeding) or χ^2^ tests (postoperative adverse events). Empty cells in the p-value column represent comparison across all categories of a variable.

**Abbreviations:** IQR, interquartile range; OR odds ratio; 95% CI, 95% confidence interval; AHI, apnea hypopnea index; AE, adverse event.

When analyzing those patients who did suffer from post-tonsillectomy hemorrhage, the hemorrhage was divided into primary hemorrhage (i.e., within 24 hours of surgery) or secondary hemorrhage (i.e., greater than 24 hours from surgery). From the five postoperative tonsillectomy hemorrhages, two (40%) were classified as primary bleeds and three (60%) were classified as secondary bleeds. Four of the post-tonsillectomy hemorrahges were from tonsillectomy performed by monopolar cautery while one was from a tonsillectomy performed by Coblator. Regarding surgical intervention, only one patient required a secondary trip to the operating room for cauterization of the post tonsillectomy hemorrhage, which occurred on postoperative day five.

Three patients with postoperative tonsillectomy hemorrhage had tonsilloliths. Indications for tonsillectomy in these patients included sleep disordered breathing (N=3), obstructive sleep apnea (N=1), and chronic tonsillitis (N=1). The remainder of the patients were discharged home within 24 hours after inpatient observation and confirmation of no recurrence of bleeding.

A post-hoc power analysis was performed as our study showed low amounts of post-tonsillectomy bleeds in regards to presence or absence of tonsilloliths. This power analysis was performed to help serve as a pilot for future prospective studies in regards to tonsilloliths and post-tonsillectomy hemorrhage. We determined that the enrollment of 817 patients would provide a power of 80% to reflect the calculated effect size as determined by this study’s incidence rates of post-tonsillectomy hemorrhage in the tonsillolith and the non-tonsillolith group at a two-sided alpha level of 0.05, making use of the two-proportion power analysis function.

## DISCUSSION

The typical rate for post-tonsillectomy hemorrhage across multiple studies range from 2.1% to 13.4%^6–15^ for which our rates are consistent with (2.7%). There is limited literature to suggest that cryptic tonsils and bacterial colonization of the tonsil are significantly associated with increased post-tonsillectomy hemorrhage, however these studies did not specify whether the tonsils contained tonsilloliths perioperatively.^17,18^ Despite the association of tonsilloliths with chronic biofilm formation and cryptic tonsils,^19,20^ our study results indicate no association between tonsilloliths and post-tonsillectomy hemorrhage. Although our sample failed to demonstrate a significant association between tonsilloliths and postoperative hemorrhage, this lack of association may be primarily attributed to the few numbers of post-tonsillectomy hemorrhages in our analytic sample.

Previous literature on tonsilloliths focuses mostly on halitosis (i.e., bad smelling breath) rather than recurrent tonsillitis.^22,24–26^ A 2014 literature review performed by Ferguson et al. investigated associations between halitosis and tonsils. This group concluded that chronic caseous tonsillitis or tonsillolithiasis was typically painless and patients can solely present with just halitosis. In addition they report that tonsilloliths are more likely to give elevated breath volatile sulfur compounds and many times they are present without clinical signs of inflammation.^22^

Recurrent or acute tonsillitis in contrast typically presents with clinical signs of infection such as fevers, sore throat, tonsillar exudates, positive strep test, and cervical lymphadenopathy.^2^ As tonsilloliths are not typically associated with these clinical inflammatory states, it may explain why we saw no significant association between presence of tonsilloliths and postoperative tonsillectomy hemorrhage.

There are mixed studies on whether or not increased BMI is associated with post-tonsillectomy hemorrhage.^27–29^ However, a 2012 study performed at the Mayo Clinic in Rochester, MN showed that severely obese children are at a significantly increased risk for perioperative airway complications.^30^ Further research is needed to identify the role that BMI plays in the presence of tonsilloliths and its clinical implications.

### Study Limitations

First, this was a retrospective cohort study limited to what was recorded in sample patients’ EHR system. Many operative reports failed to specify the presence or absence of tonsilloliths which decreased the sample size. Secondly, post-tonsillectomy hemorrhage is a relatively uncommon complication also limiting numbers. A third limitation is that our tonsillolith group was limited to the tonsilloliths that were visualized during tonsillectomy and not on histology. Tonsils that may have had deeper tonsilloliths may have been unknowingly excluded. Lastly, postoperative hemorrhage events may have been missed if a patient presented to an outside hospital and did not follow up at one of the authors’ institutions.

## CONCLUSION

In the current study we found that patients with tonsilloliths (indicating low grade chronic inflammation) had a 4.1% post-tonsillectomy hemorrahge rate compared to 1.8% of patients without tonsilloliths. However, this was not statistically significant. As there is no previous data on post-tonsillectomy bleeding and presence of tonsillolith this article can serve as a basis for further investigation on other potential predictive markers of post-tonsillectomy hemorrhage. Continued larger sample evaluations of possible risk factors for tonsillectomy are encouraged.

### Conflict of Interest

None.
